# Auditory Perception in Alport’s Syndrome

**DOI:** 10.1016/S1808-8694(15)31049-1

**Published:** 2015-10-19

**Authors:** Carla Mherlyn Viveiros, Liliane Desgualdo Pereira, Gianna Mastroianni Kirsztajn

**Affiliations:** 1 Master’s degree student in the Human Communications Disturbances Post-Graduate Program: Speech Therapy. UNIFESP. EPM (Professor); 2 Adjunct Professor, Doctor of the Speech Therapy Department at UNIFESP/EPM. (Doctoral thesis); 3 Affiliated Professor, Doctor of the Nephrology Department at UNIFESP/EPM (Doctoral thesis) SAO PAULO FEDERAL UNIVERSITY - PAULISTA MEDICAL SCHOOL (UNIFESP-EPM)

**Keywords:** otoacoustic emissions, acoustic stimulation, hereditary nephritis, auditory perception

## Abstract

Alport’s Syndrome is characterized by the presence of renal, hearing and visual disorders. Objective: To characterize the TOAE and the MOES activity (suppression effect) in individuals with Alport’s Syndrome. **Material and Method:** This is a prospective study of a sample included ten individuals with a diagnosis of Alport’s Syndrome. MOES recording was made in the presence and absence of contralateral stimulation (CLS) stimulation using the computer software ILO 92 - Otodynamics. **Results:** TOAE was present in the global response (A) and in frequency ranges of 1000, 1500, 2000 and 3000 Hz in 4 individuals (40%), and absent in 6 individuals (60%) with hearing loss. We observed no responses at 4000 Hz in the right and left ears. Individuals that presented global responses to TOAE also suppressed that response when there was noise. **Conclusion:** The suppression effect also occurs with TOAE, suggesting that the hearing loss is predominantly the result of cochlear dysfunction.

## INTRODUCTION

Hearing is important for communication between human beings in their environment. Hearing enables human beings to receive and to interpret sound information from the external milieu, and fosters language, learning and the transmission of ideas by oral and/or written communication. When hearing fails, the relation with the world of sound is compromised.

One of the genetic syndromes that include hearing loss and its consequences is Alport’s syndrome. This syndrome is characterized by renal, auditory and visual alterations.1,13

Nephritis is the most common finding,1 and usually presents in adolescence with intermittent proteinuria and/or hematuria, which progresses to renal failure. Although in most cases inheritance is X-linked (80% of cases), transmission may be heterogeneous. The frequency and severity is higher in men when inheritance is X-linked. The onset of renal failure and hearing loss may occur at any age. Similar phenotypes are expected within the same family. The dominant autossomic inheritance and autossomic recessive inheritance forms are described below, in order of frequency.

Changes may reside in a structural gene in a locus responsible for the basal glomerular membrane, the ear, and the optic capsule. This locus is responsible for the composition of collagen in the basal membrane of the aforementioned organs. In 1982 it was found that the disease occurs due to a genetic error in which certain basal membrane structures are not formed (the basal membrane remains in a fetal state). Thus, there seems to be a mutation that leads to altered production of type IV collagen, an essential component of the basal membrane in various organs.

Ophthalmologic alterations are present in 15% of patients. The most common finding is the anterior lenticonus in the lens, seen clinically as an image similar to a drop of oil in water.

Sensorineural hearing loss is typically bilateral, symmetric and progressive, starting in adolescence in 60% of men and 40% of women with Alport’s syndrome.7

Gregg and Becker8 conducted histopathology studies in 1963, which demonstrated vascular stria and ciliated cell degeneration, especially in the basal portion of the cochlea, as well as absence of the tectorial membrane.

The idea for is paper arose because we found no references in literature on the aspect of transitory otoacoustic emissions (TOAE) and activity of the medial olivocochlear efferent system (MOES) in patients with Alport’s syndrome.

The aim of this study, therefore, was to identify TOAE and MOES activity (suppression effect) in patients with Alport’s syndrome.

## MATERIAL AND METHODS

This study was carried out in the Central Auditory Processing research line of the Postgraduate program in Human Communication Disorders of the Federal University of São Paulo, approved by the ethics committee of this institution, protocol # 0240/03.

The group we studied included patients with an established diagnosis of Alport’s syndrome. Alport’s syndrome was diagnosed by the presence of typical electronic microscopic histological alterations in renal biopsies, and by the coexistence of renal histological alterations and auditory and/or ocular involvement which are common in this syndrome.

The sample included ten patients (nine men and one woman) with Alport’s syndrome, aged between 13 and 54 years. These patients had an established medical diagnosis, normal otoscopy and a bilateral type A curve tympanometry. Patients could not have other diseases or evidence of intellectual, motor or other losses, except for those pertaining to Alport’s syndrome, to be included in our sample.

A detailed medical history was made, with an emphasis on hearing-related findings. The following exams were done: pure tone audiometry, logoaudiometry, which included the speech recognition threshold (SRT) and the percentage of speech recognition loss (IPRF), measurements of acoustic immitance (tympanometry and acoustic reflex thresholds), and transitory otoacoustic emissions (TOAE), with and with no contralateral acoustic stimulation.

**Chart 1:**
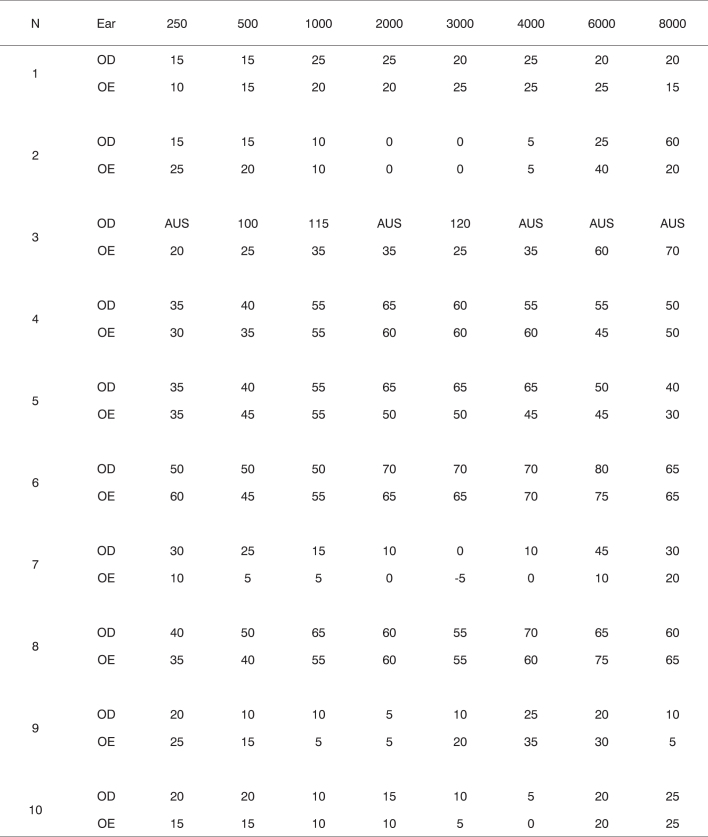
Description of the hearing thresholds by sound frequency in subjects with Alport’s syndrome, for the right (OD) and left (OE) ears.

An MA-41 MAICO audiometer was used for pure tone audiometry, SRT, and IPRF. Chart 1 shows these measurements by sound frequency.

The device AZ-7 Interacoustic was used for acoustic immitance measures (tympanometry and contralateral acoustic reflex thresholds).

TOAE with and with no contralateral acoustic stimulation allowed us to assess the function of external ciliated cells (inner ear). Contralateral acoustic stimulation was continuous white noise transmitted by the MAICO 17 audiometer, ANSI-69 standard, though a TDH-39 MX 41 headphone. Recording was made using the ILO 92 - Otodynamics software. We first recorded TOAE with no contralateral stimulation, followed by recording of TOAE with contralateral white noise at 50 dB NPS. To obtain the TOAE suppression effect value, we measured the difference between global response (A) average values without and with contralateral acoustic stimulation. We then analyzed the influence of this noise by checking whether response amplitudes were reduced or not on TOAE recordings.

We used the following non-parametric statistical tests: THE WILCOXON’S TEST, THE KRUSKAL-WALLIS TEST AND THE MANN-WHITNEY TEST. We used a 0.10 (10%) significance level in statistical comparisons. We also used the CONFIDENCE INTERVAL (95% of statistical confidence, 0.05 or 5%) technique to complete our descriptive analysis.

## RESULTS

We studied the presence or absence of global responses (A) to TOAE (in dB) with and with no contralateral acoustic stimulation by frequency range (1,000, 1,500, 2,000, 3,000 and 4,000 Hz), and the suppression effect (in dB) for the right and left ear in ten patients with Alport’s syndrome. We found that four (40%) patients had TOAE response amplitude and six (60%) did not respond (Table 1).

**Table 1:** Results of the classification of presence (P) or absence (A), for TOAE with and without noise, and for the suppression effect (in dB), for each ear in the group with Alport’s syndrome.

Subjects	TOAE WITHOUT NOISE (dB)		TOAE WITH NOISE (dB)	
	OD	OE	OD	OE
1	A	A	NR	NR
2	P (14,0)	P (14,2)	P (13,6)	P (11,7)
3	A	A	NR	NR
4	A	A	NR	NR
5	A	A	NR	NR
6	A	A	NR	NR
7	P (14,8)	P (9,7)	P (12,9)	NR
8	A	A	NR	NR
9	P (23,9)	P (20,7)	P (22,8)	P (19,0)
10	P (9,7)	P (13,9)	P (10,4)	P (13,2

NR: Not Done

P: Present

A: Absent

As a result of these findings, we analyzed the suppression effect only in patients with TOAE response amplitude.

We found that there was no statistically significant average difference between left and right ears for average values of TOAE response amplitude with no contralateral acoustic stimulation in patients with Alport’s syndrome. The TOAE response amplitude with no contralateral acoustic stimulation was seen in the global response (A) and at frequencies of 1,000, 1,500, 2,000 and 3,000 Hz (Table 2). We found no TOAE response amplitude at 4,000 Hz.

**Table 2:** Average TOAE thresholds with no contralateral acoustic stimulation in subjects with TOAE, considering the global response (A), reproducibility (R), stability (E) and response amplitude for the frequency range (in Hz) for the right ear (OD) and the left ear (OE).

EARS	A	R	E	1k	1,5K	2k	3k	4k
OD	15,60	77,50	95,50	5,67	12,33	7,25	6,50	1,50
OE	14,63	79,75	92,75	6,50	6,75	10,00	9,25	-1,00
OD X OE P-VALUE	0,715	1,000	1,000	0,593	0,109	0,109	0,144	0,317

Descriptive TOAE measurements with no contralateral acoustic stimulation were positive for the global response (A) and by frequency range at 1,000, 1,500, 2,000 and 3,000 Hz, except for 4,000 Hz (Table 3).

**Table 3:** Descriptive measurements of TOAE with no contralateral acoustic stimulation, in subjects with TOAE, considering the global response (A), reproducibility (R), stability (E) and response amplitude for the frequency range (in Hz).

INTENSITY (dB)	A	R	E	1k	1,5K	2k	3k	4k
Average (dBNPS)	15,11	78,63	94,13	6,14	9,14	8,63	7,88	0,00
Standard Deviation	4,94	16,86	5,89	10,57	8,47	4,53	3,09	2,92
Size	8	8	8	7	7	8	8	5
Minimum Value	9,7	50	80	-5	-3	2	3	-2
Maximum Value	23,9	97	98	22	20	14	12	5

We found that for the TOAE response amplitude with contralateral acoustic stimulation, there was no statistically significant difference between ears in the TOAE response amplitude with contralateral acoustic stimulation. The TOAE response amplitude with contralateral acoustic stimulation was present in the global response (A) and at 1,000, 1,500, 2,000 and 3,000 Hz, except for 4,000 Hz (Table 4).

**Table 4:** Average TOAE thresholds with contralateral acoustic stimulation in subjects with TOAE, considering the global response (A), reproducibility (R), stability (E) and response amplitude for the frequency range (in Hz) for the right ear (OD) and the left ear (OE).

EARS	A	R	E	1k	1,5K	2k	3k	4k
OD	14,93	73,75	95,25	6,33	9,33	5,25	6,00	-0,50
OE	14,63	68,25	97,00	6,67	6,67	8,25	5,00	-1,00
OD X OE P-VALUE	0,989	0,885	0,557	0,827	0,658	0,559	0,655	0,978

Analyzing descriptive TOAE measurements with contralateral acoustic stimulation, we saw that the global response global response (A) and the response at 1,000, 1,500, 2,000 and 3,000 Hz was present except at 4,000 Hz (Table 5).

**Table 5:** Descriptive measurements of TOAE with contralateral acoustic stimulation, in groups with TOAE, considering the global response (A), reproducibility (R), stability (E) and response amplitude for the frequency range (in Hz).

INTENSITY (dB)	A	R	E	1k	1,5K	2k	3k	4k
Average (dBNPS)	14,8	71,0	96,1	6,5	8,0	6,8	5,5	-0,8
Standard Deviation	4,4	21,2	2,6	7,6	5,7	6,3	2,1	3,0
Size	7	8	8	6	6	8	8	4
Minimum Value	11,5	56,3	94,3	0,5	3,4	2,4	4,1	-3,7
Maximum Value	18,1	85,7	98,0	12,5	12,6	11,1	6,9	2,2

The suppression effect is characterized by a reduction in the TOAE response amplitude with contralateral acoustic stimulation. In our study we noted the suppression effect by looking at the difference between TOAE response amplitude values with and with no contralateral acoustic stimulation. This difference was interpreted as the presence of a suppression effect, when positive, and absence thereof, when negative or absent. Furthermore, suppression was considered as positive in patients with Alport’s syndrome from a minimum average value of 0.1 dB, as this was the lowest published recorded value we found in specialized literature in audiologically normal subjects17,14,10 within the same age group as our sample.

Among the four patients with TOAE response amplitude, three had the suppression effect bilaterally; in one patient, the suppression effect was present only in the left ear (Table 6).

**Table 6:** Absence (A) and Presence (P) of the Suppression Effect of TOAE on subjects with Alport’s syndrome.

	Suppression Effect (dB)	
Subjects	OD	OE
1	NR	NR
2	0,4	2,5
3	NR	NR
4	NR	NR
5	NR	NR
6	NR	NR
7	1,9	9,7
8	NR	NR
9	1,1	1,7
10	A	0,7

NR: Not Done

A: Absent

OD: right ear

OE: left ear

Patients with Alport’s syndrome with TOAE response amplitude had an average suppression value of 0.31 dB.

We found no TOAE response amplitude at 4,000 Hz, both with and with no contralateral acoustic stimulation.

## DISCUSSION

Right and left ear responses with and with no contralateral acoustic stimulation were statistically similar. Based on these findings, we may infer that when there is TOAE amplitude response, there is adequate global cochlear function, specifically of the external ciliated cells in patients with Alport’s syndrome. This is based on Kemp’s11 (1978) report, which states that otoacoustic emissions originate in the cochlea, specifically in the external ciliated cells, which respond mechanically to auditory stimulation, depending on the normal function of the cochlear transduction process.

In our study, patients with Alport’s syndrome with TOAE response had average response amplitude values between 12 and 16 dB, within the normal range for normal adult and elderly individuals.18,9,12,6,16,4 The reduced or absent response at 4,000 Hz is also demonstrated in findings by Fenimam6 1993 and Sansone16 2000.

Therefore, we may infer that in Alport’s syndrome, the presence of TOAE occurs in line with hearing loss.

The medial olivocochlear efferent system (MOES) is responsible for keeping the basilar membrane in an optimal position for transduction, for automatic gain control for external ciliated cells, as a protection system against loud noise, and for selective attention.

The MOES activity may be verified by the TOAE suppression effect. TOAE without and with contralateral noise allows us to respectively check the function of external ciliated cells and MOES activity, which in turn allows us to establish a differential diagnosis between cochlear and retro-cochlear auditory dysfunction.

We found an average suppression effect value of 0.31 dB in our group of patients with Alport’s syndrome and TOAE, similar to those of otologically normal adults,3,10,19,17,14 and lower than average values found in studies of term newborns15,5,20 with no risk of auditory dysfunction.

The presence of suppression suggests that when activated, MOES inhibits external ciliated cell contraction, leading to reduced TOAE amplitude in otologically normal individuals. This reduction is the measurement of MOES activity. We can thus infer MOES function, and whether it was adequate in our group of patients with Alport’s syndrome, eliminating the possibility of any retro-cochlear dysfunction.

## CONCLUSION

Patients with Alport’s syndrome present TOAE results compatible with hearing loss. With TOAE there is also a suppression effect, suggesting that hearing loss is predominantly due to cochlear dysfunction.
